# Biodiversity of Phages Infecting the Dairy Bacterium *Streptococcus thermophilus*

**DOI:** 10.3390/microorganisms9091822

**Published:** 2021-08-27

**Authors:** Laurens Hanemaaijer, Philip Kelleher, Horst Neve, Charles M. A. P. Franz, Paul P. de Waal, Noël N. M. E. van Peij, Douwe van Sinderen, Jennifer Mahony

**Affiliations:** 1 DSM Biotechnology Center, 2613 AX Delft, The Netherlands; 2 School of Microbiology and APC Microbiome Ireland, University College Cork, T12 YT20 Cork, Ireland; philip.kelleher@ucc.ie; 3 Department of Microbiology and Biotechnology, Max Rubner-Institut, Federal Research Centre of Nutrition and Food, 24103 Kiel, Germany; horst.neve@mri.bund.de (H.N.); charles.franz@mri.bund.de (C.M.A.P.F.)

**Keywords:** replication, structure, phylogenetic, fermentations, cheese, food microbiology, lactic acid bacteria, phage–host interactions, recombination, modular exchange

## Abstract

*Streptococcus thermophilus*-infecting phages represent a major problem in the dairy fermentation industry, particularly in relation to thermophilic production systems. Consequently, numerous studies have been performed relating to the biodiversity of such phages in global dairy operations. In the current review, we provide an overview of the genetic and morphological diversity of these phages and highlight the source and extent of genetic mosaicism among phages infecting this species through comparative proteome analysis of the replication and morphogenesis modules of representative phages. The phylogeny of selected phage-encoded receptor binding proteins (RBPs) was assessed, indicating that in certain cases RBP-encoding genes have been acquired separately to the morphogenesis modules, thus highlighting the adaptability of these phages. This review further highlights the significant advances that have been made in defining emergent genetically diverse groups of these phages, while it additionally summarizes remaining knowledge gaps in this research area.

## 1. Introduction

*Streptococcus thermophilus* is a dominant starter bacterial species applied in global dairy fermentations, particularly in the production of yogurt and Italian and Swiss-type cheeses. The continuous and intensive application of dairy starter cultures renders them susceptible to infection by bacteriophages that may be present in the starting product, i.e., milk, or in the processing environment. A recent comparative genome analysis of 23 *S. thermophilus* strains revealed that such strains can be divided into two major clusters based on genome size, the presence or absence of *prtS*, CRISPR (clustered regularly interspaced short palindromic repeats) 2, 3 and/or 4, and the distribution patterns of restriction-modification systems, among other features [1]. This apparent homogeneity within the species creates significant opportunities for phage proliferation, while it also highlights the dependence of this species on the activity of CRISPR-Cas systems for protection against phage infection [2,3,4]. Furthermore, phage adaptation and evolutionary strategies to evade CRISPR-Cas and other barriers to infection require considerable genome plasticity, particularly considering that “natural” evolutionary processes are expedited through the industrial scale, production intensity, and consistent provision of hosts in dairy fermentation environs [5,6].

A previous study of 81 dairy streptococcal phages derived from cheese and yogurt factories identified a greater diversity among cheese-associated isolates than those isolated from yogurt, perhaps reflective of the lower diversity of strains that were applied in yogurt fermentations [7]. This study represented one of the first significant efforts to classify phages of this species and applied serological, morphological, and molecular tools to achieve this, as well as defining the host range and lifestyle of the phages (lytic versus temperate). While phage taxonomy is now highly reliant on sequence data and sequence relatedness, historical approaches to phage taxonomy were based on a range of phenotypic attributes, such as morphology and the number of major structural proteins, leading to a limited interpretation of the extent of biodiversity in phages of this species. The natural reservoir for *S. thermophilus* phages is still not precisely defined. The presence of prophages among dairy streptococci was, until recently, presumed to be low (2%), being based on prophage induction trials [7,8], and the evolutionary path of these phages remains unclear. More recently, the abundance of genome data for this species has permitted detailed genome analyses and revealed that intact prophages may be present in up to 13 % of strains, while prophage remnants may be present in a majority of strains, thereby creating a genetic reservoir for recombination with infecting virulent phages [1]. There has been an exponential increase in the number of phage genome sequences available in public databases permitting their comparison and classification. In the present review, we will (i) provide an account on the genetic and morphological diversity of the major groups of dairy streptococcal phages, (ii) discuss the role of various structural proteins in facilitating host interactions, and (iii) identify genomic regions that are associated with diversification of these phages. We highlight the major advances in dairy streptococcal phage biodiversity and classification while simultaneously reporting on gaps in our current understanding of the mechanisms that underpin the observed genomic plasticity and interactions with their hosts.

## 2. Current Taxonomy of Dairy Streptococcal Phages

### 2.1. Biodiversity and Morphological Characteristics of Dairy Streptococcal Phages

Until 2011, just two groups of dairy streptococcal phages were known to exist. These were classified based on their mode of DNA packaging, i.e., *cos* (cohesive ends) and *pac* (headful packaging method), and the number of major structural proteins that they contained [9]. These groups have recently been renamed as the *Moineauviruses* (formerly called *cos* group) and *Brussowviruses* (*pac* group) [10]. Subsequently, three additional and genetically distinct phage groups were identified, designated the 5093 [11], 987 [12,13] and the P738 [14] groups that appear to be hybrid phages or derived from phages of distinct bacterial species.

**Figure 1:**
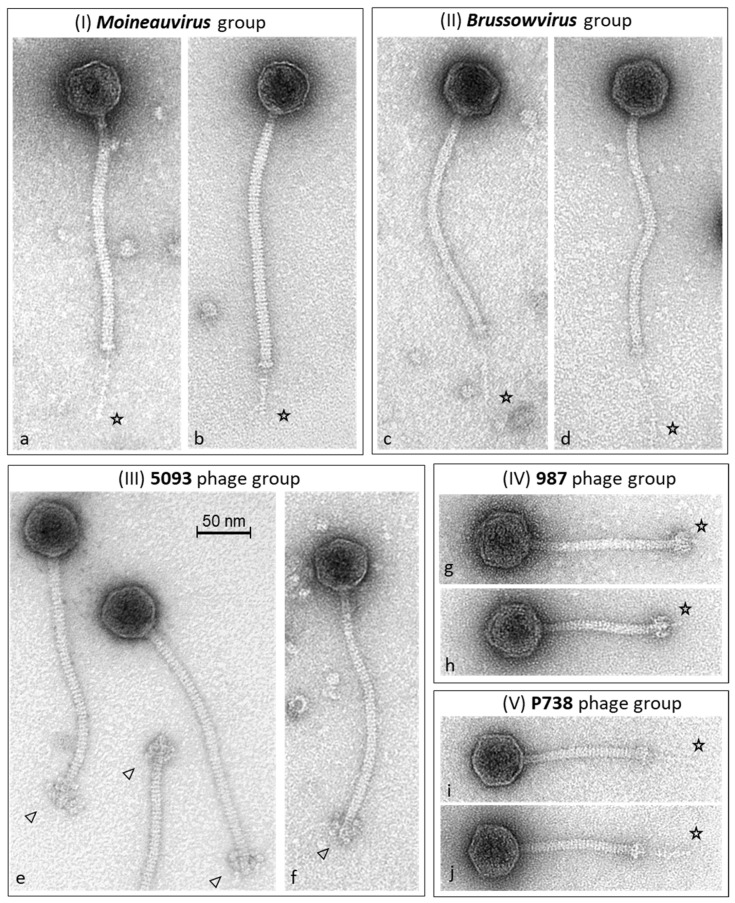
Transmission electron micrographs of negatively stained *S. thermophilus* phages representing the five phage groups (**I**) *Moineauviruses* (a. phage SW11 [16], b. phage STP1 [17]); (**II**) *Brussowviruses* (c. phage SW13 [16], d. phage CHPC1057 [18]); (**III**) 5093 phages (e. phage 5093 [11], f. phage SW27 [16]); (**IV**) 987 phages (g. phage 9871 [13], h. phage CHPC926 [18]); and (**V**) P738 phages (i. phage P738, j. phage D4446 [14]). The asterisks indicate the distal ends of the central tail fiber structures (of various lengths). The triangles show the globular appendages at the distal end of the 5093 phage tails. The (50 nm) scale bar is presented in panel e.

All dairy streptococcal phages that have been isolated to date possess long non-contractile tails and thus belong to the *Siphoviridae* family of tailed phages. Though analysis of the morphological diversity of dairy streptococcal phages by electron microscopy may not discern these phages [15], it is now timely to revisit this point as phages infecting this bacterial species are more diverse than was previously known. All phages of *S. thermophilus* exhibit isometric capsids while their tails exhibit variable lengths with distinct structures at the tail tip in certain cases. For example, the tails of members of the *Moineauvirus, Brussowvirus* and 5093 phage groups typically have a length that exceeds 200 nm, while those of the P738 and 987 groups are much shorter with lengths of 120–140 nm [11,13,14,16]. Additionally, the tail tip of members of the 987 phages possesses large appendages similar to those observed in certain lactococcal P335 phages, whereas the tail tip of virions belonging to the 5093 group contains extending globular structures (Figure 1). These distinctive features, combined with tail length observations, are now useful in identifying members of at least some of the five dairy streptococcal phage groups. However, these morphological features are not suitable to easily discern between members of the most problematic and prevalent *Moineauvirus* and *Brussowvirus* groups.

### 2.2. Genetic Diversity of Dairy Streptococcal Phages

To evaluate the robustness of the current classification system, a clustalW alignment of the deduced genomes and concatenated proteomes of 182 dairy streptococcal phages (available in the NCBI database) commencing at the small terminase subunit protein (and following the genomic order) was performed. All protein sequences of the 182 phages were included. The alignment was employed to generate a phylogenetic tree using the “iTOL” software (http://itol.embl.de/) (accessed on 2 April 2021), applying the neighbor-joining method. This analysis confirmed the overall classification of all assessed phages among one of five major genotypes of *S. thermophilus* phages (A representation of the phylogenetic tree based on the whole nucleotide sequences is presented in Figure S1), being consistent with a recent analysis of 100 dairy streptococcal phage genomes at nucleotide level [14]. It further highlights that most sequenced phages currently described are classified among the *Moineauvirus* species (61% or 111 of 182 analyzed) or *Brussowviruses* (25% or 46 of 182 phages analyzed). These statistics, based on genome analysis, compare well to a recent survey of an industrial collection of *S. thermophilus* phages using PCR-based typing methods in which the *Moineau-* and *Brussowviruses* were observed to represent the dominant genotypes in a collection of 1179 phages (69% and 29%, respectively) [19].

While the overall genome/proteome analysis supports the existence of five distinct phage groups, perhaps what is less clear is the presence of shared functional modules between distinct groups that may unravel the evolutionary pathway of phages of this bacterial species. It has been noted that some dairy streptococcal phages represent hybrids of (pro)phages of distinct bacterial host species. For example, the 987 group phages are suggested to represent genomic hybrids containing a replication module similar to the *Moineauvirus*, 7201, and a morphogenesis-associated module with similarity to lactococcal P335 phages (being consistent with the associated morphological features, which will be discussed later) [13]. Similarly, the P738 phages (P738 and D4446) appear to be genetic hybrids containing a limited number of dairy streptococcal phage structural protein-encoding genes (believed to be involved in host interactions) and a replication module that is derived from non-dairy streptococcal phages including those of *Streptococcus pyogenes* [14]. In addition to these hybrids at host species level, it is also worth mentioning that several reports of module shuffling and DNA exchange between dairy streptococcal phage groups have been published. For example, the replication module of certain 5093 group phages was observed to share homology to those present in members of the *Brussowvirus*, *Moineauvirus* and the 987 group [12,13,20]. Similarly, a significant level of sequence relatedness was observed across the replication modules of the *Moineauvirus* P7573 and the *Brussowvirus* P7572. The region encoding the tail structural components, which include elements presumed to be involved in host interactions, is also similar between these phages (P7572 and P7573), highlighting the significant plasticity of these phage genomes [20]. This mosaicism is not a new concept and, in fact, was highlighted in 1999 in a comparative genome analysis of five *S. thermophilus* phages [21]. This early observation of insertions and deletions across the genomes of these phages has been corroborated in recent studies and allows us to now investigate the possible source of such divergences, thereby providing insights into their evolutionary pathway(s).

**Figure 2:**
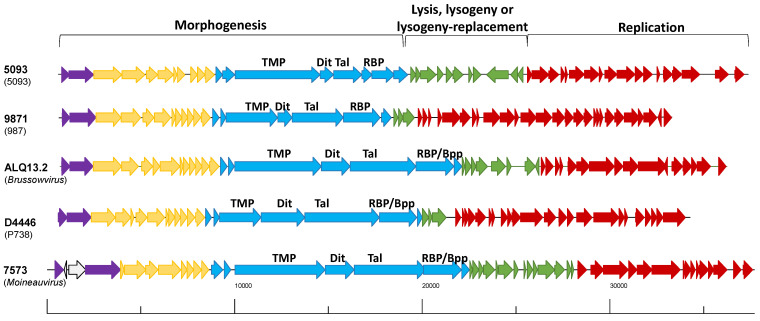
Schematic depicting the genome organization of representatives of each of the five dairy streptococcal phage groups, i.e., 5093 (5093 group); 9871 (987 group); ALQ13.2 (*Brussowvirus*); D4446 (P738 group); and 7573 (*Moineauvirus*). Each arrow represents a gene, and the arrows are colour-coded to identify the functional module to which they belong, i.e., the morphogenesis module encompassing DNA packaging (purple), capsid morphogenesis and head-to-tail joining (yellow) and tail morphogenesis (blue) functions; the lysis and lysogeny or lysogeny-replacement module (green) and DNA replication (red). The genomes are drawn according to the scale indicated below the schematic.

To ascertain the modular diversity and removing the bias of overall genome content, we selected 25 phages representing a spread across observed clusters in the overall proteomic tree (Figure S1) and representing those where previous observations of modular shuffling had been reported (Moineauviruses Sfi19, Sfi21, 9A, 128, Abc2, DT1, SW6, V2, STP1, CHPC572, P7573 and 7201; Brussowviruses 2972, Sfi11, P7572, ALQ13.2, TP-J34, P7955, and O1205; P738 phages P738 and D4446; 5093 phages 5093 and P0092; and 987 phages 9871 and SW22). Since these 25 phages are spread across the phylogenetic tree based on overall nucleotide content, we considered these to be representative of the overall cohort of dairy streptococcal phages. Secondly, the genomes were considered to contain three major gene clusters, i.e., those involved in replication, morphogenesis, and lysogeny (Figure 2). The morphogenesis modules were considered to encompass the genomic region associated with encoding proteins involved in DNA packaging and capsid/tail morphogenesis. Between the morphogenesis modules and the presumed replication modules are genes whose encoded products are predicted to be involved in host lysis and lysogeny. Lysogeny and so-called “lysogeny replacement modules” appear to be universally present among dairy streptococcal phages [21] and are highly diverse. The replication modules were arbitrarily defined as all genes downstream of those encoding lysis/lysogeny (replacement) functions and including those with identifiable DNA replication functions (Figure 2). A major defining feature of the 5093 and P738 phage groups is the genetic novelty associated with the structural modules among dairy streptococcal phages. Furthermore, the replication modules of dairy streptococcal phages have recently been reported to fall into one of two major groups [20]. Since these two functional modules are present in all dairy streptococcal phage genomes and with at least some conserved elements to allow comparison, they were selected as the basis of a focused genetic analysis. A phylogenetic analysis of the structural proteomes and replication proteomes (and excluding the lysogeny/lysogeny replacement modules) as distinct modules was undertaken to ascertain the diversity of modules that may act as a reservoir for the evolution of dairy streptococcal phages.

#### 2.2.1. Replication Modules Display Inter-Group Relatedness

**Figure 3:**
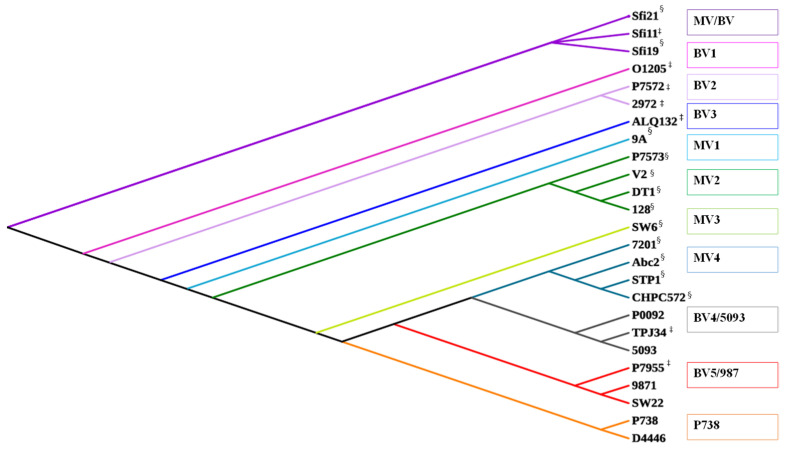
Proteomic tree of the replication proteomes of 25 representative phages. *Moineauvirus*-specific replication modules are colored light blue (MV1), mid-blue (MV4), dark green (MV2) and light green (MV3). *Brussowvirus*-specific replication modules are highlighted in bright pink (BV1), lilac (BV2), and dark blue (BV3). A module that is shared among certain members of both *Brussow-* and *Moineauvirus,* 5093 or 987 members is indicated in deep purple (MV/BV), grey (BV4/5093) or red (BV5/987), respectively. The replication module proteome of P738 is unique and represented in orange. ^§^ denotes Moineauviruses; ^‡^ denotes Brussowviruses; the 5093 phages are represented by 5093 and P0092; the 987 group are represented by 9871 and SW22; and the P738 group are represented by P738 and D4446.

Significant genetic diversity was observed among the replication modules of the 25 analyzed phages; however, members of distinct phage groups were regularly observed to harbor at least one protein with sequence similarity above 50 % (Figure 3). Based on this analysis, there appear to be four *Moineauvirus*-specific replication modules (designated MV1 through to MV4) and five *Brussowvirus*-specific replication modules (named BV1-5) as well as one replication module type that is shared between members of both phage groups (called MV/BV), notwithstanding that the groups are arbitrarily established based on the cladogram presented in Figure 3. Additionally, one *Brussowvirus* replication module bears similarity to that of 5093 and another that is shared with 987 phages (Figure 3). These observed genetic “overlaps” are suggestive of evolution/emergence of the 5093 and 987 groups through recombination with temperate *Brussowviruses*. Interestingly, the replication module of phage P7955, a *Brussowvirus* that was previously reported to possess a unique replication module (i.e., that did not align with *Brussowvirus* O1205 or *Moineauvirus* 7201) according to Stanley et al. [22]), clusters with those of the 987 phages (Figure 3). While it is not possible to discern which is the ancestral phage lineage, it is tempting to speculate that the *Brussowvirus* group is likely the ancestral phage, given their prevalence in industrial fermentation environs for several decades. Phages P738/D4446 are confirmed as being unique among dairy streptococcal phages within the replication module.

#### 2.2.2. Morphogenesis Modules Are Highly Group-Specific

**Figure 4:**
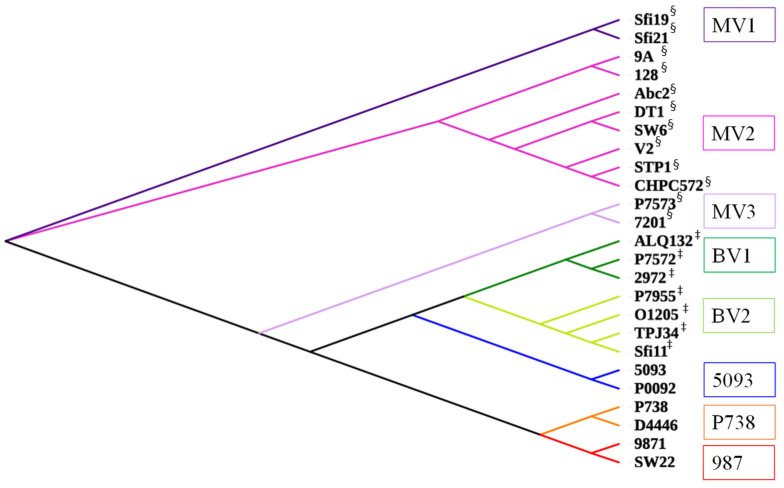
Proteomic tree highlighting distinct morphogenesis modules of the analyzed phages. Pink and purple clades and text boxes highlight those specific to *Moineauvirus* members (MV1, MV2 and MV3). Those colored light and dark green (BV1 and BV2) highlight *Brussowvirus*-specific morphogenesis modules; in blue is the single 5093-like module; orange relates to the P738-specific module and red indicates the 987-specific module. ^§^ denotes Moineauviruses; ^‡^ denotes Brussowviruses; the 5093 phages are represented by 5093 and P0092; the 987 group are represented by 9871 and SW22 and the P738 group are represented by P738 and D4446.

Based on the analysis of 25 representative phage morphogenesis modules, it was observed that the modules clustered well according to the five major groups with subgroups observed among the *Moineauvirus* (MV) and *Brussowvirus* (BV) modules (Figure 4). Interestingly, the 5093-like morphogenesis module aligned most closely with those of the Brussowviruses, while those of the P738 and 987 phage groups formed separate clades on the tree (Figure 4). Based on this analysis, three *Moineauvirus* (MV1-3) and two *Brussowvirus* (BV1-2) phylogroups of structural modules could be discerned. Interestingly, the MV3 morphogenesis module appeared to be phylogenetically closer to BV1 than the MV1 and MV2 modules, which may represent an evolutionary link between the *Moineauvirus* and *Brussowvirus* modules. Furthermore, the 5093 phages and *Brussowvirus* phages morphogenesis modules cluster closely in the phylogenetic tree, highlighting a likely historical evolutionary link between these groups.

### 2.3. Evolutionary Pathways of Dairy Streptococcal Phages

Based on the phylogenetic analysis of the replication and morphogenesis modules of 25 representative dairy streptococcal phages, eleven and eight clusters of replication and morphogenesis module types, respectively, were arbitrarily identified. To assess the number of modular arrangements present among the analyzed phage proteomes, the derived proteome types of the morphogenesis and replication modules were collated (Table 1). Sixteen combinations of replication and morphogenesis modules were identified using this approach. While many of the possible combinations of replication and morphogenesis modules present in currently sequenced phage genomes may be represented among the sixteen combinations described in this review, it is likely that as additional phage genomes are sequenced, that this number will rise considerably. The sample group of 25 phages highlighted the high level of diversity of the currently sequenced phage genomes and the combinations of replication and morphogenesis modules. Among the Moineauviruses, seven combinations of the structural and replication modules were observed with all except one module being unique to the Moineauviruses (Sfi19 and Sfi21-like; See Table 1). As mentioned above, certain Brussowviruses possess shared genetic content with phages of the 5093 and 987 groups, and this is reflected in the diversity of combinations observed among the *Brussowvirus,* 5093, and 987 phage genomes (Table 1). Finally, the P738 group appears to be unique among dairy streptococcal phages and likely evolved separately from all other dairy streptococcal phage groups.

Currently available sequence data are heavily biased in favor of the *Moineauvirus* and *Brussowvirus* members as these have been studied at the sequence level since the 1990s, being in stark contrast to the more recently identified phage groups. However, based on current data, two dominant *Moineauvirus* (MV)-specific morphogenesis modules exist (MV1 and MV2). These MV-specific modules appear to recombine preferentially with replication modules of other *Moineauvirus* phage genomes and, to a lesser extent, with certain *Brussowvirus* members (Table 1). In contrast, there is a greater prevalence of *Brussowvirus*-related replication modules among members of four of the five phage groups, indicating that *Brussowvirus* members contribute significantly to the overall evolution of dairy streptococcal phages possibly through recombination with temperate members.

## 3. Interactions of Dairy Streptococcal Phages and Their Hosts

### 3.1. Phage Adhesion Device

The interactions of dairy streptococcal phages with their hosts are known to be multi-factorial, encompassing carbohydrate binding domains that are present in structural proteins associated with the distal tail structure which is termed the adhesion device [23,24,25]. The adhesion device incorporates (part of) the tail tape measure protein (TMP), the distal tail protein (Dit), the tail-associated lysin (Tal) and a receptor binding protein (RBP) in the case of the 5093 and 987 phages. In addition, phages belonging to the *Moineauvirus*, *Brussowvirus,* and P738 groups possess an additional and fully conserved structural protein (referred to as Bpp or Tal+1). While the role of variable domains within the so-called Tal protein has long since been established [23], additional structural proteins have also been implicated in phage binding by model phages, such as the *Moineauvirus* DT1 [24]. The variable regions (VR) of Tal have been implicated in the recognition and binding to the host cell of the *Moineauvirus* DT1, and VR2 sequences have been observed to largely correlate with host strain irrespective of phage group, i.e., *Moineauvirus* or *Brussowvirus*; for example, it was observed that phages with distinct host ranges may also group, suggesting that other structural components are additionally involved in the binding process [19]. Furthermore, the Dit of many analyzed *Moineauvirus* and *Brussowvirus* members are “evolved”, implying that they contain a carbohydrate binding domain, while the 987 and 5093 phage members harbor classical Dits, being devoid of such domains. For a detailed review of the domain architecture of the adhesion device proteins of dairy streptococcal phages, see [25]. Structural homology analysis tools are essential for accurate functional annotations of proteins, particularly in the absence of sequence homology. Here, we analyzed the Bpp amino acid sequences of 22 representatives of *Moineauvirus*, *Brussowvirus* and P738 phages, as well as the RBP of representative 5093 and 987 group phages using the structural prediction software HHpred [26].

**Figure 5:**
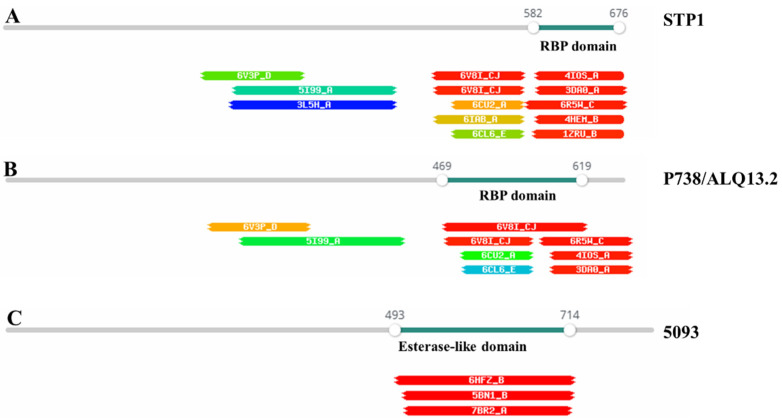
Sample outputs of HHpred analysis of representative (**A**) *Moineauvirus* STP1 Bpp, (**B**) *Brussowvirus* (ALQ13.2) and P738 phage (P738) Bpp, and (**C**) 5093 phage-encoded RBP, highlighting the distinct specificities and architectures of the adhesion device proteins of these phages. Within each protein “bar” a protein domain is highlighted in green where high probability of sequence/structural conservation is observed. The protein domains with sequence/structural similarity are presented below each bar. For the identified protein domain of STP1 (**A**), residues 582–676 of its Bpp represent a TP901-1 RBP (PDB 4I0S_A) domain with high probability (>97%). Similarly, the Bpps of P738 and ALQ13.2 (**B**) possess a RBP domain similar to that of the staphylococcal phage phi80 alpha (PDB 4I0S_A). The RBP of 5093 (**C**) possesses an esterase-like domain similar to that of *Roseburia intestinalis* (PDB 6HFZ_B; probability >97%). Other domains with reduced probability are also observed; however, deductions of likely function are affiliated with that yielding the highest probability “hit” as described above.

The RBP of 9871 (987 group) encompasses an N-terminal domain with structural similarity to the upper baseplate protein (BppU) of the lactococcal P335 phage TP901–1 (4V96_AQ with 99.8% probability) and with an extended C-terminal region that has previously been reported to bear similarity to the *Bacillus* phage Phi29 RBP [25]. This protein architecture is reminiscent of the lactococcal P335 phage RBP subgroup II that appear to have a fused “upper baseplate” and receptor binding protein [27]. The 987 phages recognize an exopolysaccharide (EPS) receptor on the host cell surface [28]. Not all *S. thermophilus* strains produce EPS, and the loci associated with their biosynthesis are diverse [19,29], two key factors that underpin the specificity of the 987 phages. The 5093 phage RBP has previously been reported to exhibit an esterase-like domain at the C-terminal end of the protein (Figure 5) [25]. Several saccharidic moieties in the Gram-positive cell wall (EPS, CWPS, and teichoic acids) may incorporate (phosphodi)ester-linked components; thus it is plausible that the esterase domain is functional with respect to one or more of these cell wall polysaccharides [30,31]. All analyzed Bpps encoded by *Moineauvirus* and *Brussowvirus* members harbor RBP domains with structural relatedness to those of the lactococcal phage TP901–1 (PDB 4I0S_A), *Listeria* phage PSA (6R5W_C), and/or *Staphylococcus* phage phi80 alpha (C), with at least 97% probability (Figure 5). Therefore, it is assumed that the Bpp of these phages performs the RBP function and acts as the primary determinant of host-recognition specificity, an activity that is enhanced and supported by host-binding activities of carbohydrate binding domains present in Dit and/or Tal proteins, where appropriate. This proposal is supported by the observation of higher binding affinity/fluorescent labelling of the Bpp carbohydrate binding domain compared to that of Tal host cells of the *Moineauvirus* STP1 [24]. For detailed reading on the structural proteins involved in phage–host interactions, please see [19,25].

### 3.2. RBP Phylogeny Is Not Necessarily Linked to That of the Overall Morphogenesis Module

**Figure 6:**
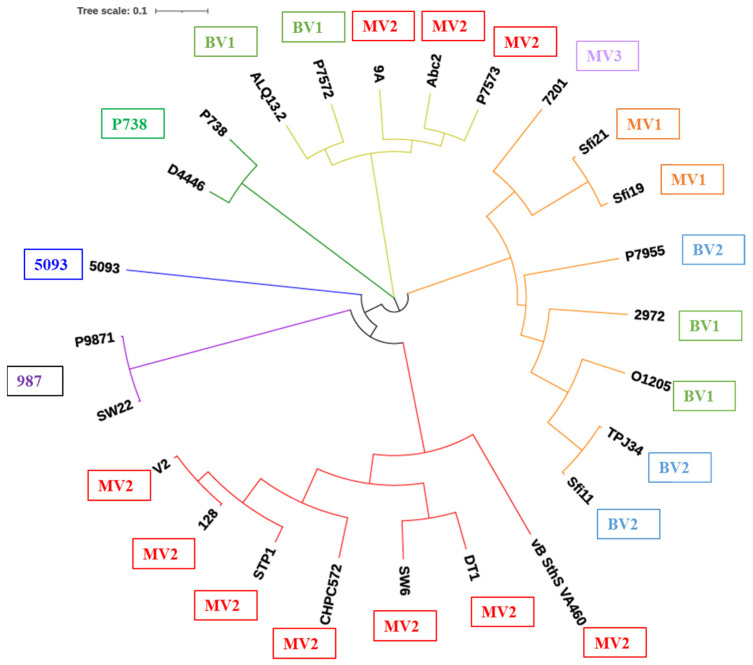
Phylogenetic tree based on the amino acid sequence alignment of carbohydrate binding domain sequences of the RBP or Bpp of 25 dairy streptococcal phages. The colored clades are based on the phage groups to which the phages belong, i.e., P738, dark green; *Brussowvirus* members, lime green; *Moineauvirus* members, orange or red; 987, purple; 5093, deep blue. The text boxes outside the tree indicate the groupings of morphogenesis modules identified in Figure 4, Table 1. There is a distribution of binding domain sequence types among *Brussowvirus* and *Moineauvirus* members, suggesting that the binding domains have been acquired separately to the overall morphogenesis modules in certain phages.

To develop an understanding of the diversity of binding modules within the encoded RBPs or Bpps of the 25 representative phages and to determine if they evolved with the overall morphogenesis modules, an alignment of the identified binding domains was performed. Multiple sequence alignments of the deduced amino acid sequences of the RBP/Bpp carbohydrate binding domains, whose boundaries were identified using HHpred analysis described in the previous section and typically representing the C-termini of the respective proteins, were performed using ClustalW software. The alignment was employed to generate an unrooted phylogenetic tree using the “iTOL” software (http://itol.embl.de/) (accessed on 26 April 2021), applying the neighbor-joining method. This analysis suggests that the RBPs have not necessarily co-evolved with the overall morphogenesis modules, as the RBP/Bpp host binding domain phylogenetic groupings do not necessarily correlate with the overall morphogenesis module phylogroups presented in Table 1 (Figure 6). The identified phylogenetic groups show that there is considerable overlap between RBP binding domains encoded by members of the *Moineauvirus* and *Brussowvirus* groups, supporting the role of this domain in host specificity rather than being linked with the genetic lineage of the phage. It also highlights the genetic plasticity of this genomic region and is congruent with the observation of evolved structural proteins, including Dit and Tal proteins, among dairy streptococcal phages [14,25]. Such genomic plasticity facilitates the incorporation of multiple or alternative carbohydrate (or receptor) binding domains, thereby enhancing the binding affinity/avidity, or altering/expanding the host range of the phage.

The modular nature of phage RBPs and module shuffling within these proteins has been demonstrated in the development of a lactococcal phage TP901–1 chimera harboring the RBP head domain of the *Skunavirus* p2 [32]. Therefore, this shuffling appears to be an evolutionary (universal) strategy among phages to allow rapid adaptation to available hosts in their environment to ensure their continued success [33]. Further evidence of genomic plasticity and modular shuffling is provided by the P738 phages (P738 and D4446) [14]. The P738 phage genomes bear little sequence similarity to other dairy streptococcal phages with the exception of two genes within the morphogenesis module that encode structural proteins associated with host interactions [14]. Furthermore, these phages harbor a Bpp-like protein that possesses the same RBP domain observed among the assessed *Moineauvirus* and *Brussowvirus* phages (Figure 5 and Figure 6). Therefore, dairy streptococcal phage adhesion appears to require multiple carbohydrate-binding proteins, of which the Bpp of *Moineauvirus*, *Brussowvirus* and P738 groups apparently represents a *bona fide* RBP (Figure 5), which in some cases is supported by carbohydrate-binding activities of Tal- and Dit-associated carbohydrate binding activities [25].

## 4. Conclusions

The ever-increasing number of available genome sequences of *S. thermophilus* phages has been transformative to our understanding of the diversity and interactions of these phages with their hosts. The observation of hybrid phage genomes and extensive genome plasticity appears to be widespread among phages of this bacterial species. While five major groups of dairy streptococcal phages are currently identified, the extent of module shuffling highlighted in this review underscores the need for consistent monitoring of dairy fermentation facilities, as they represent evolutionary melting pots that facilitate the emergence of new phages. *Moineauvirus* and *Brussowvirus* undoubtedly remain the most persistent threat to thermophilic dairy fermentations; however, the repeated presence of 987 and 5093 phage members in recent studies [11,12,13,16,20] suggests that recently discovered phage groups may become more problematic in coming decades if the corresponding hosts are applied with increased intensity. It also seems likely that additional “hybrid” phage groups will emerge in the years to come, further endorsing the need for continuous monitoring of phage populations in industrial fermentations.

Recent studies aimed at identifying the receptor for dairy streptococcal phages have identified saccharidic moieties including rhamnose-glucose polysaccharides and exopolysaccharides as the primary receptor for several phages [18,28,34]. However, significant knowledge gaps remain in this area, and an improved functional examination of the primary determinants of phage binding will enhance our understanding of the key determinants of phage evolution in the coming decade, just as knowledge of the CRISPR-Cas (and phage-encoded anti-CRISPR) systems has informed and guided our understanding of dairy streptococcal phage evolution over the past decade [2,4,5,6,35,36,37]. Bioinformatic tools, such as HHpred, combined with increasingly populated protein structure and genome sequence databases, will continue to strengthen the predictive abilities and interpretations and will facilitate improved protein annotations. In the present review, we identified apparent module shuffling events between dairy streptococcal phage species in terms of their replication and morphogenesis modules; however, there do not appear to be limitations or obvious recombination “hotspots” within these modules apart, perhaps, from the adhesion device module. Within this region of the morphogenesis module, phages appear to acquire carbohydrate binding or host-specifying modules to enhance their adaptive potential to available host(s). In this review, the role of members of *Brussowvirus* species, many of which are temperate (pro)phages has been proposed as an influencer and source of dairy streptococcal phage evolution. Increasing numbers of complete streptococcal genomes are becoming available each year and with sufficient data pertaining to the prophages of this species, the mechanism that underpins this evolutionary process may become evident.

## Figures and Tables

**Table 1:** Summary of replication and morphogenesis module types based on structural and replication module phylogenetic analysis.

Phage	Phage Group	Replication Module Type	Morphogenesis Module Type
9A	*Moineauvirus*	MV1	MV2
V2, DT1, 128	*Moineauvirus*	MV2	MV2
P7573	*Moineauvirus*	MV2	MV3
SW6	*Moineauvirus*	MV3	MV2
STP1, CHPC572, Abc2	*Moineauvirus*	MV4	MV2
7201	*Moineauvirus*	MV4	MV3
Sfi19, Sfi21	*Moineauvirus*	MV/BV	MV1
Sfi11	*Brussowvirus*	MV/BV	BV2
O1205	*Brussowvirus*	BV1	BV2
P7572, 2972	*Brussowvirus*	BV2	BV1
ALQ13.2	*Brussowvirus*	BV3	BV1
TP-J34	*Brussowvirus*	BV4/5093	BV2
P7955	*Brussowvirus*	BV5/987	BV2
9871, SW22	987	BV5/987	987
5093, P0092	5093	BV4/5093	5093
P738, D4446	P738	P738 (*S. pyogenes* phage T12)	P738

## Supplementary Materials

The following is available online at https://www.mdpi.com/article/10.3390/microorganisms9091822/s1, Figure S1: Phylogenetic tree based on the complete nucleotide sequences of 182 dairy streptococcal phages available in the NCBI database. Members of the *Moineauvirus* (purple branches), *Brussowvirus* (pale blue branches), 5093 (green branches), 987 (orange branches) and P738 (red branch) are represented in this analysis. Phage names highlighted in bold face blue text are those representatives selected for further analysis of their respective morphogenesis and replication modules (D4446 was also selected as the only other representative of the P738 group).
