# Supplementary figures and images for: Biodiversity of Phages Infecting the Dairy Bacterium Streptococcus thermophilus
Source: Microorganisms. 2021 Aug 27;9(9):1822. doi: 10.3390/microorganisms9091822 (PMC8470116; doi:10.3390/microorganisms9091822)

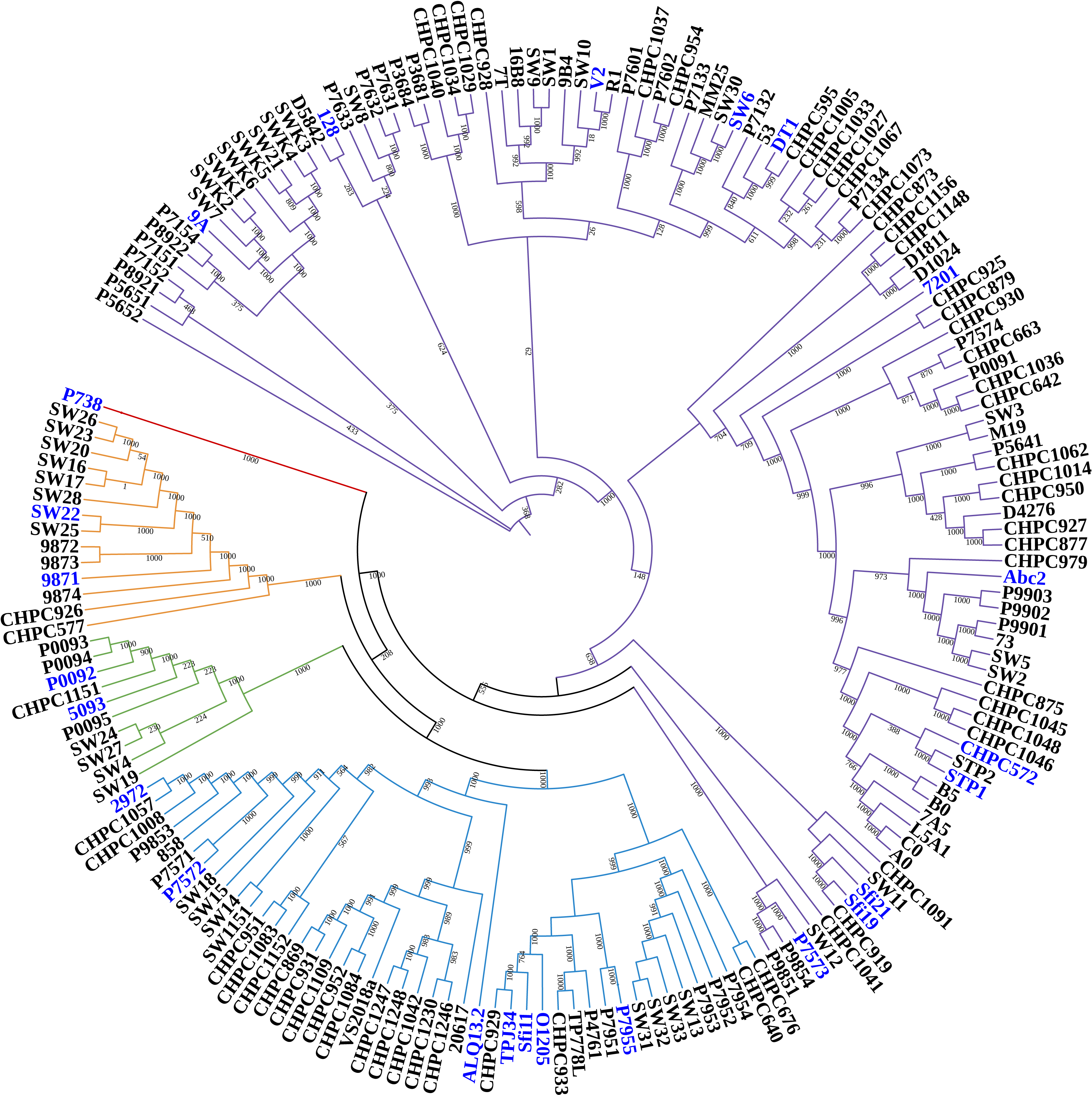

Supplement: Supplementary file 1 [file microorganisms-09-01822-s001.zip › microorganisms-1330656-supplementary.tif]
